# Phenotypic variability of Niemann-Pick disease type C including a case with clinically pure schizophrenia: a case report

**DOI:** 10.1186/s12883-018-1124-2

**Published:** 2018-08-17

**Authors:** Tomoya Kawazoe, Toshiyuki Yamamoto, Aya Narita, Kousaku Ohno, Kaori Adachi, Eiji Nanba, Atsuko Noguchi, Tsutomu Takahashi, Masamitsu Maekawa, Yoshikatsu Eto, Masafumi Ogawa, Miho Murata, Yuji Takahashi

**Affiliations:** 1 Department of Neurology, National Center Hospital, National Center of Neurology and Psychiatry, 4-1-1 Ogawahigashi, 187-8551, Kodaira, Tokyo, Japan; 2 Division of Child Neurology, Institute of Neurological Science, Tottori University Faculty of Medicine, Yonago, Tottori Japan; 3 Division of Functional Genomics, Research Center for Bioscience and Technology, Tottori University, Yonago, Tottori Japan; 4 Department of Pediatrics, Akita University Graduate School of Medicine, Akita, Akita Japan; 5 Department of Pharmaceutical Sciences, Tohoku University Hospital, Sendai, Miyagi Japan; 6 Advanced Clinical Research Center, Institute for Neurological Disorders, Kawasaki, Kanagawa Japan

**Keywords:** Niemann-Pick disease type C, Schizophrenia, Dystonia, Miglustat, *NPC1* mutation

## Abstract

**Background:**

Niemann-Pick disease type C (NPC) is a lysosomal storage disorder with severe prognosis. Disease-specific therapy is crucial to prevent disease progression; however, diagnosing NPC is quite difficult because of remarkably variable clinical presentations. The NPC Suspicion Index (NPC-SI) was developed to overcome this problem. Identifying preclinical cases is important for prevention and therapy. Here, we report three newly diagnosed NPC cases, one typical juvenile-onset case and the cases of two sisters with symptoms neurologically/psychiatrically indistinguishable from dystonia and schizophrenia, respectively.

**Case presentation:**

In Case 1, a 25-year-old man presented with a 14-year history of intellectual disability, clumsiness, spastic ataxia, dysphagia, and frequent falls. Neurological examination revealed vertical supranuclear gaze palsy and involuntary movements. Ultrasonography revealed mild splenomegaly, and filipin staining of skin fibroblasts was positive with a variant staining pattern. *NPC1* gene analysis showed compound heterozygous mutations, including c.1421C > T (p.P474L), a known causative mutation, and c.3722 T > C (p.L1241S), a new mutation. In Case 2, a 28-year-old woman, the proband, who had marked splenomegaly in her childhood, survived well, contrary to the expected severe prognosis of infantile NPC. She had minor neuropsychiatric symptoms including auditory hallucinations, nocturnal urination, and sleep paralysis. At the age of 28 years, she presented with a 1-year history of orofacial and oromandibular painful dystonia. The patient’s 35-year-old sister (Case 3) was diagnosed with schizophrenia. In both cases, filipin staining of skin fibroblasts was positive with variant staining patterns, as well as elevated levels of urinary bile acids. *NPC1* gene analysis showed compound heterozygous mutations including c.3011C > T (p.S1004 L), a known causative mutation, and c.160_161insG (p.D54GfsX4), a new mutation. Their mother, who was under therapy with modafinil for narcolepsy, shared the latter mutation.

**Conclusions:**

Marked clinical variability was observed in our three cases. NPC could masquerade as a pure neuropsychiatric disorder such as dystonia or schizophrenia. Abdominal ultrasonography, history evaluation, and neurological examination were quite important in the diagnostic process.

## Background

Niemann-Pick disease type C (NPC, MIM# 257220) is an autosomal recessive lysosomal storage disease caused by *NPC1* (95%) or *NPC2* (5%) gene mutations. If untreated, the prognosis of NPC is severe, with most patients with infantile-, juvenile-, and adult-onset NPC dying by the age of 10 years, before the age of 30 years, and in their mid-30s, respectively [1].

Disease-specific therapies with miglustat [2] or intrathecal 2-hydroxypropyl-β-cyclodextrin [3] are available; therefore, early and accurate diagnosis is critical. However, establishing the diagnosis of NPC is challenging because of its clinical variability. In order to facilitate diagnosis, the NPC Suspicion Index (NPC-SI) was developed, which includes seven key discriminatory features: splenomegaly, neonatal jaundice, gelastic cataplexy, vertical supranuclear gaze palsy (VSGP), cognitive decline, psychotic symptoms, and family history [4]. NPC-SI assists in the identification of neurologically advanced cases with the key clinical features; however, especially in adult cases, an initial diagnosis of psychiatric illness, such as schizophrenia or depression, is usually made and NPC diagnosis is delayed by several years until the appearance of neurological complications [5]. Here, we report three cases of NPC; one juvenile-onset sporadic case and two adult sister cases. The latter two were diagnosed at clinical stages with minimum neurological complications, resulting in early therapeutic interventions.

## Case presentation

The case profiles are summarized in Table 1.

**Table 1:** Case profiles

	Case 1	Case 2	Case 3
Age at onset (years)	11	27	22
Initial symptom	Intellectual disability	Painful dystonia	Schizophrenia
Age at diagnosis (years)	25	28	35
Visceral signs			
Splenomegaly	Mild	Mild	Mild
Neonatal jaundice	+	None	None
Neurological signs			
VSGP	+	None	None
Dysphagia	+	None	None
Spastic ataxia	+	None	None
IVM	Chorea, athetosis, dystonia, myoclonus	Dystonia	None
Psychiatric signs			
Cognitive decline	+	None	None
Psychotic symptoms	+	+	+
NPC-SI ^a^			
Percentile	98%	18%	32%
RPS	183	47	67
Filipin pattern	Variant	Variant	Variant
ASM activity	221	213	165
(nmol/mg protein/h)	99% of control	95% of control	73% of control
Phenotype (cDx)	Juvenile	Adult	Adult
*NPC1* gene mutations			
Father	c.1421C > T	c.3011C > T	c.3011C > T
	(p.P474L)	(p.S1004 L)	(p.S1004 L)
Mother	c.3722 T > C	c.160_161insG	c.160_161insG
	(p.L1241S)	(p.D54GfsX4)	(p.D54GfsX4)
Brain MRI	Sl. atrophic	Normal	Sl. atrophic
Hypoperfusion on ECD-SPECT	Frontal	Frontal	Frontal
CSF Analysis			
p-Tau (< 55)	59 pg/mL	47 pg/mL	36 pg/mL
h-Tau (< 300)	371 pg/mL	214 pg/mL	188 pg/mL
Cupper			
Serum Cu (> 68)	76 μg/dL	82 μg/dL	88 μg/dL
Serum Cp (> 21)	22 mg/dL	18 mg/dL	22 mg/dL
Urine Cu (> 20)	NA	NA	32 μg/day
Urine bile acid ^b^			
SNAG-Δ5-CT (< 11)	NA	13 ng/mL	92 ng/mL
SNAG-Δ5-CA (< 282)	NA	42 ng/mL	317 ng/mL
SNAG-Δ5-CG (< 258)	NA	69 ng/mL	481 ng/mL

Abbreviations: *ASM* Acid sphingomyelinase; *cDx* Clinical diagnosis; *Cp* Ceruloplasmin; *CSF* Cerebrospinal fluid; *Cu* Copper; *ECD-SPECT* Technetium-99 m ethyl cysteinate dimer-single photon emission computed tomography; *h-Tau* Human tau protein; *IVM* Involuntary movements; *MRI* Magnetic resonance imaging; *NA* Not assessed; *NPC-SI* Niemann-Pick disease type C-suspicion index; *p-Tau* Phosphorylated tau protein; *RPS* Risk prediction score; *VSGP* Vertical supranuclear gaze palsy

a) Hendriksz CJ, et al. J Rare Disord. 2015 [4]. b) Maekawa M, et al. Steroids. 2013 [7]

### Case 1

**Fig. 1:**
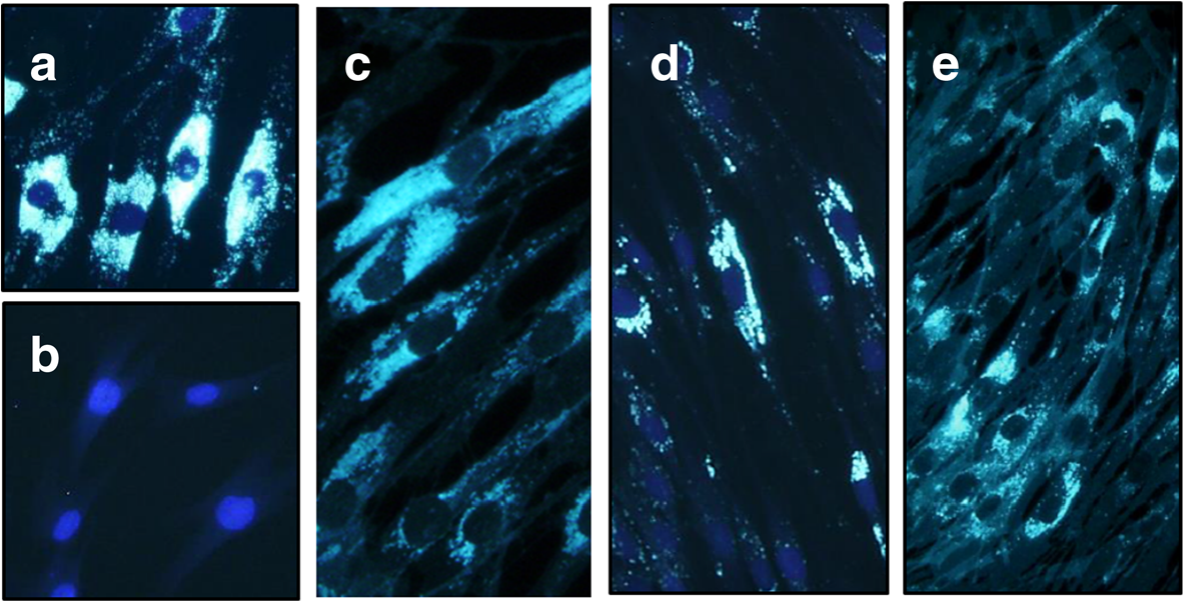
Filipin staining of skin fibroblasts. **a** Positive typical (classical) control from a patient with Niemann-Pick disease type C. **b** Negative control from a healthy volunteer. All cases were positive with a variant staining pattern. **c** Case 1. **d** Case 2. **e** Case 3

A 25-year-old man presented with a 14-year history of intellectual disability (since 11 years of age), clumsiness (12 years), spastic ataxia (16 years), slow and slurred speech (17 years), schizophrenic delusions (18 years), dysphagia (19 years), and frequent falls (21 years). He had prolonged neonatal jaundice, for which exploratory laparotomy and simultaneous cholecystectomy were performed at that time. He was also diagnosed with Crohn’s disease at the age of 16 years. Neurological examination at presentation revealed VSGP and involuntary movements including choreoathetosis, dystonia, and myoclonus. Ultrasonography revealed unpalpable mild splenomegaly. The NPC-SI was 98% with a risk prediction score (RPS) of 183 (high likelihood of NPC when the NPC-SI is more than 14% and the RPS is more than 40). Filipin staining of skin fibroblasts showed a variant pattern (Fig. 1). Gene analysis of *NPC1* using genomic DNA extracted from the patient’s blood via Sanger sequencing revealed compound heterozygous mutations, including the known c.1421C > T (p.P474L) mutation [6] from the father and a novel c.3722 T > C (p.L1241S) mutation from the mother. We deposited the latter mutation to the Leiden Open Variation Database (LOVD); the individual number of this data entry is 00165102. Oral intake of miglustat for 12 months did not relieve his symptoms. The treatment had to be discontinued because the patient had succumbed to a vegetative state following massive pneumonia secondary to severe ileus due to Crohn’s disease.

### Case 2

The proband, a 28-year-old woman, presented with a 1-year history of orofacial and oromandibular dystonic movements. The woman had no neonatal jaundice; however, at the age of 1 year, marked hepatomegaly (2 cm) and splenomegaly (7 cm) were noted. Vacuolated lymphocytes were observed in her blood and cerebrospinal fluid, and many foam cells were observed in the bone marrow. The enzymatic activity of acid sphingomyelinase in skin fibroblasts had decreased to 17 nmol/mg/h (118 ± 53). As there was relatively conserved enzymatic activity (more than 10% of control), a diagnosis of infantile-onset NPC was considered. However, contrary to the expected severe prognosis of known infantile cases, she survived well with minimal neurological symptoms. The patient developed auditory hallucinations, nocturnal urination, and sleep paralysis at the ages of 3, 6, and 9 years, respectively. These symptoms gradually subsided by the age of 12 years. She had an eating disorder between the ages of 17–19 years, and excessive daytime sleepiness appeared at the age of 19 years. Orofacial and oromandibular dystonic movements with facial pain appeared at the age of 27 years. Modafinil 300 mg/day was not effective for sleepiness, and medications such as amitriptyline 10 mg/day, zonisamide 100 mg/day, trihexyphenidyl 2 mg/day, and L-dopa 50 mg/day were not effective for the painful dystonia. Only clonazepam 2 mg/day minimally relieved the symptoms. At the age of 28 years, when she was referred to our hospital, she was neurologically intact except for the dystonia. Ultrasonography detected mild unpalpable splenomegaly. The NPC-SI was 18% with an RPS of 47. Filipin staining of the skin fibroblasts was positive with a variant staining pattern (Fig. 1). The urinary levels of bile acids [7] were partially elevated (Table 1). *NPC1* gene analysis showed compound heterozygous mutations including the known c.3011C > T (p.S1004 L) mutation [8] from the father and a new mutation of c.160_161insG (p.D54GfsX4) from the mother. Oral intake of miglustat for more than 12 months was not effective for the painful dystonia.

### Case 3

The elder sister of the patient in Case 2 was clinically diagnosed with schizophrenia, with good response to antipsychotic medications and was later found to have NPC. She was examined at the age of 8 years, when her younger sister was suspected to have NPC; however, she was neurologically intact with no splenomegaly at that time. By the age of 22 years, she began showing signs of schizophrenia; an official diagnosis confirmed the disease at the age of 25 years. She responded well to risperidone 3 mg/day. When we examined her at the age of 35 years, she was neurologically intact. Moreover, in this Case, ultrasonography detected mild unpalpable splenomegaly. Given the family history of Case 2, the NPC-SI was 32% with an RPS of 67. *NPC1* gene analysis detected the same mutations with those observed in Case 2. We deposited the new mutation to LOVD; the individual numbers are 00165175 (Case 2) and 00165176 (Case 3). Oral intake of miglustat for 20 months improved her psychotic symptoms, and her attending psychiatrist diagnosed complete remission of schizophrenia.

## Discussion and conclusions

This case series highlights that patients with NPC may exhibit remarkable phenotypic variability, which in our cases ranged from juvenile-onset progressive neuropsychiatric deterioration to adult psychiatric symptoms clinically indistinguishable from schizophrenia. The phenotypic variability is further supported by previous studies reporting that a substantial number of patients with NPC do not exhibit cardinal symptoms such as VSGP and dysphagia [9, 10]. Phenotypic heterogeneity was even observed in a case of monozygotic twins [11]. Meanwhile, the international study on genetic screening for psychiatric patients with neurological symptoms or splenomegaly, known as the ZOOM study, demonstrated that 3 of 250 participants harbored pathogenic mutations in *NPC1* [12]. Of note, the patient in Case 3 did not show any neurological symptoms at the time of NPC diagnosis. Therefore, the phenotypes of NPC must be more diverse than previously thought, and patients with mild clinical manifestations may remain undiagnosed. Based on these observations, we would like to emphasize that NPC should be considered in the differential diagnosis of a wide variety of disease entities, including juvenile-onset multi-systemic neurological disorders and adult psychiatric disorders such as schizophrenia.

It is of vital importance to accurately diagnose NPC because disease-modifying therapies are available. Furthermore, it is imperative to diagnose it in the early stages, particularly in cases with mild neurological symptoms, because of the severe prognosis of adult NPC, which results in death at a mean age of 38 years [9] or 33 years [10], and the difficulty in ameliorating the neurological symptoms even with miglustat, when the disease is at an advanced stage. In contrast, the appearance of psychotic symptoms consistent with schizophrenia or depression precedes the appearance of neurological complications by several years in more than half of the cases [13], and the effectiveness of miglustat in alleviating the psychotic symptoms [14] has been reported, further corroborating the importance of early detection. For this purpose, the NPC-SI was invented and revised based on the cardinal features of NPC [4]. The NPC-SI in this study varied greatly, depending on the extent to which the key features were detected at the time of diagnosis. The NPC-SI showed that there was high likelihood of NPC in all cases, with the recommendation that the patients should be referred to the NPC referral center (institute) for immediate testing, highlighting the clinical usefulness of the NPC-SI. The fact that the mean RPS of adult cases was as high as 179 [15] implies that most cases were diagnosed at a stage of fully present clinical manifestations. To benefit from the NPC-SI, it is imperative to enhance the awareness of its usefulness in daily clinical settings, particularly in adult neurology.

In Japan, the current initial diagnostic workup for NPC includes a combination of serum oxysterol (7-ketocholesterol) and urinary bile acids, followed by more specific diagnostic procedures including skin biopsy with filipin staining and *NPC1* or *NPC2* gene analysis. In our cases, mild elevation of urine bile acids in Cases 2 and 3 was informative in the initial workup, whereas the oxysterol value in Case 2 was normal (could not be tested in Cases 1 and 3). Additionally, in line with previous reports, cerebrospinal tau proteins were elevated in Case 1 [16] and the laboratory values of copper metabolism slightly deviated or were close to the lower limit of the normal levels [17]. Recently, it has been reported that plasma oxysterol (cholestane-3β,5α,6β-triol) is informative in the detection of NPC [18]. Additionally, lysosphingolipids have been proposed as a potential biomarker for NPC [19]. Establishing excellent biomarkers for NPC is highly warranted considering that skin biopsy and genetic analyses are time-consuming and expensive procedures.

Finally, we would like to focus on the narcolepsy experienced by the mother of Cases 2 and 3, who shared the c.160_161insG (p.D54GfsX4) mutation. In addition to gelastic cataplexy as one of the key discriminatory features of NPC, sleep problems, such as excessive daytime sleepiness, are known to be related to NPC, presumably via dysfunction of the locus coeruleus [20]. Regarding partial manifestations in people with *NPC1* gene heterozygosity, a previous report of a family whose proband developed late infantile NPC with compound heterozygous mutations of *NPC1* described that the heterozygous asymptomatic carriers of *NPC* mutations, the oldest sister and parents, had foam cells in their bone marrow smears [21]. Likewise, it is possible that the haploinsufficiency of the *NPC1* gene identified in our pedigree could also cause mild tissue changes resulting in manifesting carriers. Accumulation of familial histories and genetic evidence is necessary to analyze the relationship between narcolepsy and *NPC1* mutation carriers. We hypothesize that *NPC1* might be an extremely rare candidate gene for familial narcolepsy syndrome.

In conclusion, the cases described in this report highlight the clinical variability of NPC and reinforce the importance of early diagnosis of this rare disease to initiate disease-modifying therapy. We would like to emphasize that the exploration of disease-suggestive findings by use of the NPC-SI in patients with schizophrenia would enhance the diagnostic accuracy of NPC.

## Abbreviations

NPC: Niemann-Pick disease type C
NPC-SI: NPC suspicion index
RPS: Risk prediction score
VSGP: Vertical supranuclear gaze palsy
